# The N-terminal domain of the thermo-regulated surface protein PrpA of *Enterococcus faecium* binds to fibrinogen, fibronectin and platelets

**DOI:** 10.1038/srep18255

**Published:** 2015-12-17

**Authors:** Ana M. Guzmán Prieto, Rolf T. Urbanus, Xinglin Zhang, Damien Bierschenk, C. Arnold Koekman, Miranda van Luit-Asbroek, Janneke P. Ouwerkerk, Marieke Pape, Fernanda L. Paganelli, Dominique Wobser, Johannes Huebner, Antoni P. A. Hendrickx, Marc J. M. Bonten, Rob J. L. Willems, Willem van Schaik

**Affiliations:** 1 Department of Medical Microbiology, University Medical Center Utrecht, Utrecht, The Netherlands; 2 Department of Clinical Chemistry and Hematology, University Medical Center Utrecht, Utrecht, The Netherlands; 3 Division of Infectious Diseases, University Medical Center Freiburg, Freiburg, Germany; 4 Division of Pediatric Infectious Diseases, Hauner Children´s Hospital, Ludwigs Maximilian University Munich, Munich, Germany

## Abstract

*Enterococcus faecium* is a commensal of the mammalian gastrointestinal tract, but is also found in non-enteric environments where it can grow between 10 °C and 45 °C. *E. faecium* has recently emerged as a multi-drug resistant nosocomial pathogen. We hypothesized that genes involved in the colonization and infection of mammals exhibit temperature-regulated expression control and we therefore performed a transcriptome analysis of the clinical isolate *E. faecium* E1162, during mid-exponential growth at 25 °C and 37 °C. One of the genes that exhibited differential expression between 25 °C and 37 °C, was predicted to encode a peptidoglycan-anchored surface protein. The N-terminal domain of this protein is unique to *E. faecium* and closely related enterococci, while the C-terminal domain is homologous to the *Streptococcus agalactiae* surface protein BibA. This region of the protein contains proline-rich repeats, leading us to name the protein PrpA for proline-rich protein A. We found that PrpA is a surface-exposed protein which is most abundant during exponential growth at 37 °C in *E. faecium* E1162. The heterologously expressed and purified N-terminal domain of PrpA was able to bind to the extracellular matrix proteins fibrinogen and fibronectin. In addition, the N-terminal domain of PrpA interacted with both non-activated and activated platelets.

Enterococci are Gram-positive, facultative anaerobic bacteria that are ubiquitously present in nature and which can grow in a wide range of temperatures between 10 °C and 45 °C^1^. The genus *Enterococcus* comprises around forty different species, including *Enterococcus faecium* and *Enterococcus faecalis*^2^. These two species are common commensals of the mammalian gastrointestinal tract, but in the last decades they have become important causes of nosocomial infections, ranging from urinary tract infections to infective endocarditis^3^.

In the late 1970s, *E. faecalis* was responsible for almost 95% of the *Enterococcus*-associated infections, with *E. faecium* being a comparatively rare cause of disease. In the last three decades this pattern has shifted, and now *E. faecium* is becoming an increasingly frequent cause of hospital-associated infections^4^,^5^. Furthermore, the infections caused by *E. faecium* are often more difficult to treat than those caused by *E. faecalis.* This is due to the antibiotic resistance determinants that have recently accumulated in nosocomial *E. faecium* strains and which confer resistance to clinically important antibiotics, including β-lactams and vancomycin^6^.

*E. faecium* hospital-acquired bloodstream infections are often associated with the use of invasive medical devices such as catheters and implants, which disrupt the continuity of the epithelium. Notably, patients with a bacteremia caused by *E. faecium* have a worse prognosis than patients with bacteremias caused by other enterococci^7^,^8^. Bacterial surface-exposed proteins which can potentially interact with exposed host tissues, for instance due to the use of indwelling medical devices, have been studied in *E. faecium* to clarify the interactions of the bacterial cell with its host^9^. Several *E. faecium* surface proteins that are anchored to the peptidoglycan through a motif composed of leucine-proline-X-threonine/serine/alanine-glycine^10^,^11^, have been identified and characterized. These include pili^12^,^13^, surface proteins involved in biofilm formation^14^,^15^ and proteins that recognize the components of the extracellular matrix (ECM)^10^, including microbial surface components recognizing adhesive matrix molecules (MSCRAMMs)^16^,^17^.

Platelets, which are normally responsible for maintaining the balance in blood between fluidity and coagulation^18^, can contribute to binding of bacteria to host tissues which promote disease^19^. Gram-positive bacteria can attach to platelets either through direct binding of bacterial surface proteins to platelets^20^,^21^,^22^ or by indirect interactions mediated through plasma molecules like fibrinogen and fibronectin^23^. Fibrinogen and fibronectin are not only essential in the coagulation cascade for the clotting of blood, but can also be found as constituents of the host ECM^24^,^25^. In enterococci, several proteins have previously been described to interact with fibrinogen and fibronectin^26^,^27^,^28^, but only the Ebp-type pilus of *E. faecalis* has been shown to have a role in adherence to platelets^29^.

In this study we aimed to identify and characterize novel *E. faecium* proteins that may contribute to host-cell interactions. In order to successfully establish colonization or an infection, commensal and pathogenic bacteria need to adapt to the conditions that prevail in the host. Temperature is an important environmental signal that is frequently linked to the differential expression of bacterial virulence genes^30^,^31^. Consequently, we first performed comparative transcriptome profiling of *E. faecium* growing at 25 °C and 37 °C, to identify genes that exhibited higher expression at mammalian temperatures. We subsequently focused on a gene, encoding a surface protein (termed PrpA), that exhibited higher expression at 37 °C than at 25 °C. PrpA was further characterized in terms of its capacity to bind to ECM components and platelets.

## Results

### Thermo-regulated expression of a gene encoding a previously uncharacterized *E. faecium* surface protein

**Figure 1:**
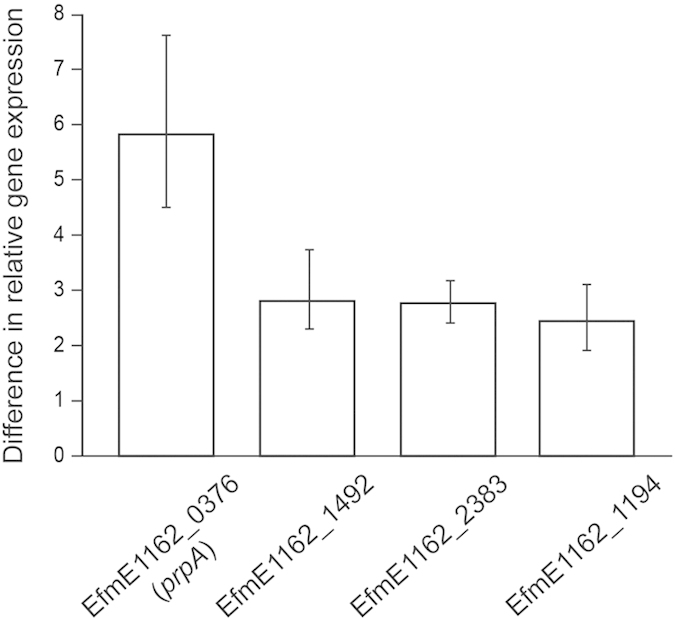
Differences in relative gene expression of *E. faecium* E1162 between 25 °C and 37 °C. Expression levels of four genes exhibiting higher expression (in the transcriptome analysis) during mid-exponential growth at 37 °C compared to 25 °C were determined by qRT-PCR. The data from the qRT-PCR were normalized using *tufA* as housekeeping gene. The differences in gene expression between 37 °C and 25 °C are shown. Error bars correspond to the standard deviation of four biological replicates.

To identify *E. faecium* genes which exhibit thermo-regulated gene expression and consequently may have a role in virulence or colonization, we performed a transcriptome analysis of *E. faecium* E1162 during mid-exponential growth at 25 °C and 37 °C. In the transcriptome analysis, the differences in gene expression observed between 25 °C and 37 °C were relatively limited, reflecting the homeostatic nature of enterococcal physiology under permissive growth conditions. Nevertheless, thirty-three genes showed significantly higher expression at 37 °C compared to 25 °C. Supplementary Table 1 shows the genes that were up-regulated at 37 °C. The transcriptome analysis results were confirmed by qPCRs, in which we measured the expression of the four genes that exhibited the largest fold-difference in their expression at 37 °C versus 25 °C, as determined by the microarray analysis (Fig. 1). One of the genes that were differentially regulated during growth at 37 °C (4.4-fold, compared to growth at 25 °C), is predicted to encode a surface protein (locus-tag: EfmE1162_0376). The up-regulated expression of EfmE1162_0376 at 37 °C, combined with the observation that surface-exposed proteins frequently have a role in host-bacterial cell interactions in enterococci and other Gram-positives^11^,^32^, led us to further study the function of EfmE1162_0376.

After PCR amplification, cloning and sequencing of EfmE1162_0376 from *E. faecium* E1162 in an overexpression construct (see below), we noticed that the sequence that was originally deposited to Genbank for *E. faecium* E1162 (accession number: EFF35774) was inaccurate as one of the repeats in EfmE1162_0376 was lacking. Presumably, DNA repeats in the coding sequence of EfmE1162_0376 could not be correctly resolved using the pyrosequencing technology that was previously used to sequence the *E. faecium* E1162 genome^33^. The corrected sequence of EfmE1162_0376 has been deposited to Genbank (accession number: KF475784). EfmE1162_0376 (which was previously designated *orf884* by Hendrickx *et al.*^34^) is present ubiquitously in *E. faecium* strains from different environments, including bloodstream infections and feces of hospitalized patients, healthy individuals and farm animals^34^.

**Figure 2:**
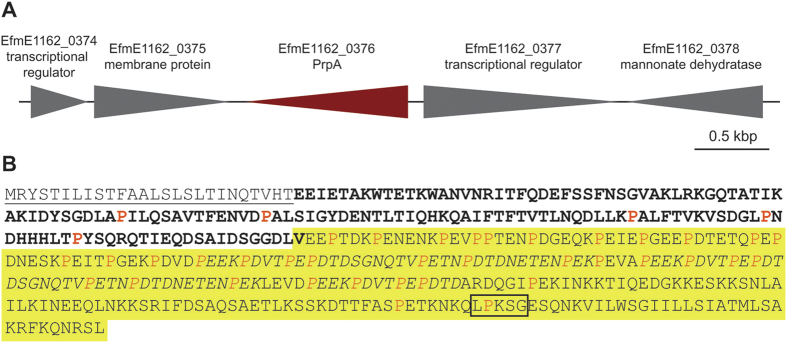
The genetic context of *prpA* and overview of the PrpA protein. Panel A shows the genetic environment of the *prpA* gene (in red). Arrows indicate the direction of transcription. The amino acid sequence of the PrpA protein is shown in Panel B. The N-terminal signal sequence is underlined and the LPxSG-motif is boxed. The position of the overexpressed and purified PrpA_27-167_ protein fragment is indicated in bold. The region of the protein that is highlighted in yellow is homologous to BibA of *S. agalactiae* (further details in the text) and repeats in this region are in italics. Proline residues in PrpA are indicated in orange.

The genes that flank EfmE1162_0376 are transcribed in opposite directions, suggesting that EfmE1162_0376 is transcribed monocistronically (Fig. 2A). An overview of the protein encoded by EfmE1162_0376 is provided in Fig. 2B. The N-terminal domain of the protein encoded by EfmE1162_0376 contains a signal sequence, which is predicted to be required for the targeting of the protein to the cell wall. Based on currently available genome sequences, orthologous domains of the N-terminal region of this protein only exist in *E. faecium* and closely related species, like *E. hirae, E. mundtii* and *E. durans*, but not in *E. faecalis* or other bacteria. The C-terminal part of EfmE1162_0376 contains a predicted LPxTG-type anchor (LPKSG), which is expected to be required for the cell-wall anchoring of the protein to peptidoglycan by sortases^32^. Furthermore, the C-terminal part contains repeat regions that are rich in proline, aspartic acid and glutamic acid residues. The high number of proline residues (41 of 370 amino acids in the surface-anchored protein) present in the protein led us to name the protein PrpA, for proline-rich protein A. The C-terminal region of PrpA is similar (35% amino acid identity) to the C-terminal repeat motif of BibA, an adhesin of *Streptococcus agalactiae*^35^. Like in BibA, the proline-rich repeats of PrpA might serve to protrude the N-terminal domain to the outside.

The N-terminal domain of surface exposed proteins is of primary importance for their function in enterococci^36^,^37^ and other gram positive bacteria^38^. Therefore, we decided to focus on the role of the of the previously uncharacterized N-terminal domain of PrpA and its ability to interact with host components.

### Determination of the transcriptional start site of *prpA*

**Figure 3:**
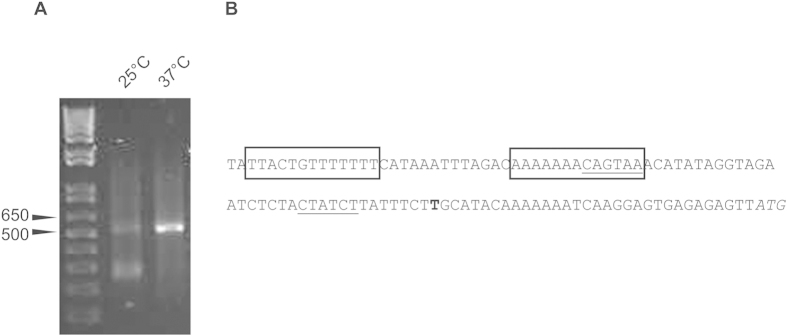
Mapping of the transcriptional start site of *prpA* by 5′ RACE. (**A**) Agarose gel electrophoresis of 5′ RACE PCRs shows the product at approximately 520 bp obtained during 5′RACE after nested amplification of cDNA (reverse transcribed from RNA isolated at 25 °C and 37 °C). (**B**) The upstream region of the *prpA* gene is shown. The transcriptional start site determined by 5′ RACE is indicated in bold. The boxes indicate the inverted repeats that are discussed in the text. Putative −35 and −10 promoter regions are underlined. The ATG start codon of *prpA* is shown in italics.

In order to better understand the mechanism of the thermo-regulation of *prpA* expression, we performed 5’ rapid amplification of cDNA ends (5′ RACE) to map the 5′-end of *prpA* and to identify the promoter region of *prpA*. Upon gel electrophoresis of the 5′ RACE reactions, a band was observed at approximately 520 bp in the reaction that was performed with RNA isolated at 37 °C (Fig. 3A). The 5′ RACE reaction performed on RNA isolated during mid-exponential growth at 25 °C was considerably weaker, perhaps reflecting the lower expression of *prpA* at this temperature. Cloning and sequencing of the product revealed the transcriptional start site of *prpA* (Fig. 3B). Upstream of the *prpA* promoter, we identified two inverted repeats that may play a role in the transcriptional regulation of *prpA*.

### PrpA is a surface protein that is most abundant during exponential growth at 37 °C

**Figure 4:**
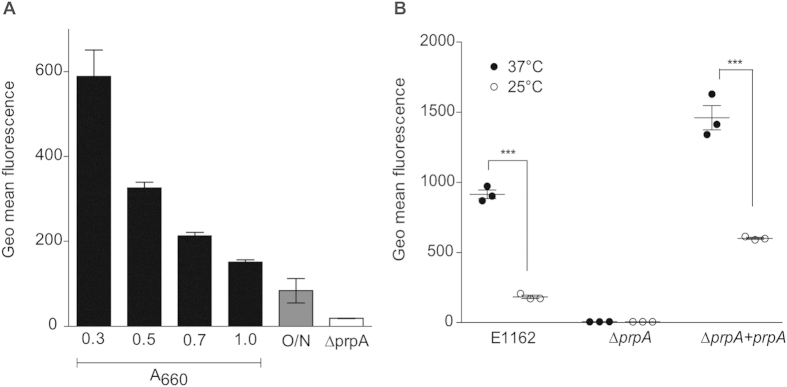
Analysis of surface-exposed PrpA levels. PrpA was detected on the surface of the bacterium by flow cytometry using rabbit anti-PrpA IgG and FITC-labeled goat anti-rabbit IgG. Panel A shows PrpA-levels on the surface of wild-type *E. faecium* E1162 grown at 37 °C. Samples were taken throughout exponential phase (A_660_ 0.3, A_660_ 0.5), transition phase (A_660_ 0.7) and early stationary phase (A_660_ 1.0). Levels of surface-exposed PrpA in an overnight culture of E1162 and during mid-exponential growth of ∆*prpA* are also shown. Panel B shows levels of surface-exposed PrpA in *E. faecium* E1162, ∆*prpA* and ∆*prpA* + *prpA* at 25 °C and 37 °C during mid-exponential growth (A_660_ 0.3). The data presented were obtained in three independent experiments and significant differences (*p* < 0.01, Student’s t-test) are indicated by three asterisks. The geometrical mean of the fluorescence detected in the FITC channel is shown on the y-axis in both panels.

**Figure 5:**
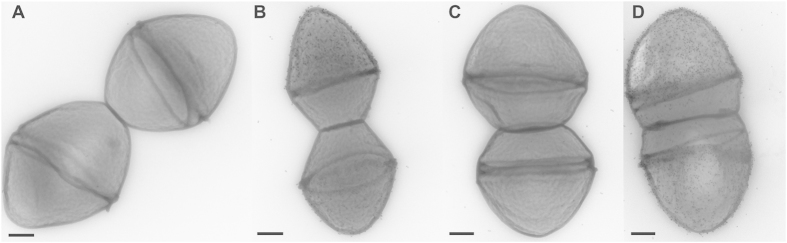
Cellular localization of PrpA assessed by transmission electron microscopy. *E. faecium* E1162, ∆*prpA* and ∆*prpA* + *prpA* were grown at 37 °C to A_660_ 0.3. Panel A shows cells incubated with pre-immune serum (negative control). Figure B to D show the localization of PrpA in the wild type, ∆*prpA* and complemented strain, respectively. PrpA was detected using rabbit anti-PrpA IgG and a subsequent incubation with 10 nm protein A-gold beads. The scale bar corresponds to 200 nm.

In order to further characterize the function of the N-terminal fragment of PrpA, we heterologously overexpressed and purified PrpA_27–167_, corresponding to the N-terminal domain of PrpA, minus the signal sequence. We also constructed a markerless *prpA* mutant (*ΔprpA*) and a strain in which the mutation was complemented *in trans* (*ΔprpA* + *prpA*). No growth defect was observed in the mutant and complemented strain compared to the E1162 wild-type (Fig. S1A and S1B) when grown at 25 °C and 37 °C. Subsequently, we quantified surface-exposed levels of PrpA in exponential, stationary phase and overnight cultures of E1162 at 37 °C by flow cytometry, using polyclonal anti-PrpA antibodies. Levels of surface-exposed PrpA were highest in E1162 during early stages of exponential growth, when cells were harvested at A_660_ = 0.3 during growth at 37 °C. Quantities of surface-exposed PrpA declined at later stages of growth (Fig. 4A). Additionally, we measured the production of PrpA in E1162, *ΔprpA* and *ΔprpA* + *prpA* at 25 °C and 37 °C. Since we observed the highest levels of surface-exposed PrpA in E1162 at A_660_ = 0.3, we chose this condition for our subsequent experiments. In Fig. 3B we show that cells of E1162 grown at 25 °C had significantly lower levels of PrpA at the surface than cells grown at 37 °C, indicating that the lower expression of *prpA* at 25 °C observed in the transcriptome analysis translates to lower levels of surface-associated PrpA protein. As expected, in *ΔprpA* no signal was detected on the surface of cells. PrpA production is restored in the complemented strain, but also in this strain, levels of PrpA are higher at 37 °C than at 25 °C. Subsequently, we used transmission electron microscopy (TEM), to visualize the surface localization of PrpA in *E. faecium*, using polyclonal anti-PrpA antibodies and a protein A-10 nm gold particle conjugate (Fig. 5A,D). TEM showed that PrpA localized to the ‘old’ hemispheres of dividing cells of E1162 (Fig. 5B) and of *ΔprpA* + *prpA* (Fig. 5D). No apparent change in cellular morphology due to either the deletion or the overexpression of *prpA* was observed.

### Thermo-regulation of PrpA is observed in other *E. faecium* strains

**Figure 6:**
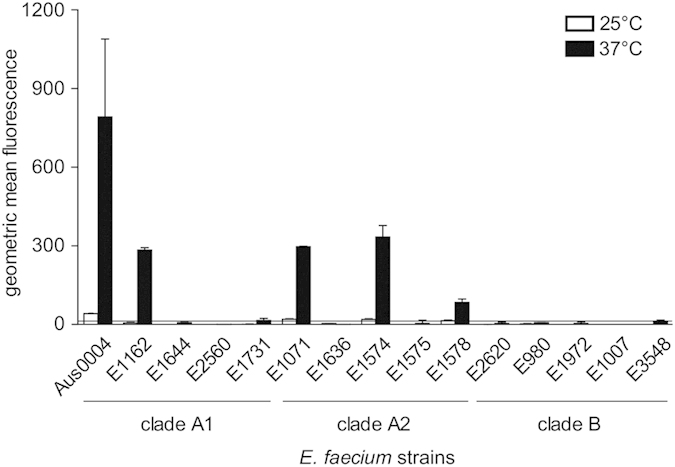
Production of PrpA in *E. faecium* strains. Production of PrpA is shown in fifteen different *E. faecium* strains isolated from diverse environments and belonging to different clades of the *E. faecium* species. Bacteria were grown to mid-exponential phase at either 25 °C or 37 °C and PrpA production was determined by flow cytometry. White and black bars indicate the production of PrpA at 25 °C and 37 °C, respectively. The horizontal line indicates the signal obtained from ∆*prpA*, where no PrpA is produced. Error bars indicate the standard deviation of the results in three independent experiments.

After characterizing the temperature-dependent production of PrpA in strain E1162, we studied the production of PrpA in fourteen *E. faecium* strains, of which the genomes were previously sequenced and which together covered the different phylogenetic clades of *E. faecium*, i.e. clade A1 representing clinical isolates, clade A2 representing animal strains and clade B representing human commensals^39^. All tested strains contained a copy of the *prpA* gene that was ≥79% identical to *prpA* in *E. faecium* E1162, with the difference mainly being introduced through the variable number of proline-rich repeats. We found that none of the clade B strains produced PrpA during mid-exponential growth (A_660_ = 0.3) at 25 °C or at 37 °C (Fig. 6). Besides E1162 (a clade A1 strain), we found that one additional strain from clade A1 and three strains from clade A2, were able to produce PrpA when grown at 37 °C. Interestingly, in all these strains the production of PrpA was found to be thermo-regulated, with higher levels at 37 °C, as observed before for E1162, when compared with 25 °C (Fig. 6). In the strains (*n* = 10) in which we were not able to demonstrate production of PrpA at the cell surface, sequence analysis of the *prpA* gene revealed that four strains (E2560, E1575, E2620 and E1007) contained mutations in *prpA* that led to a truncation of the gene.

Alignment of the *prpA* promoter regions of the clade A strains did not provide evidence to why E1644, E1731 and E1636, which contain an intact copy of *prpA,* failed to produce this protein. The promoter sites, including the inverted repeat upstream of *prpA* promoter in *E. faecium* E1162, were completely identical to all of these three strains. In contrast to clade A strains, the same region in clade B strains contained several SNPs, which may affect the transcriptional regulation of the *prpA* gene in the clade B strains (Fig. S2).

### PrpA_27-167_ binds to fibrinogen and fibronectin

**Figure 7:**
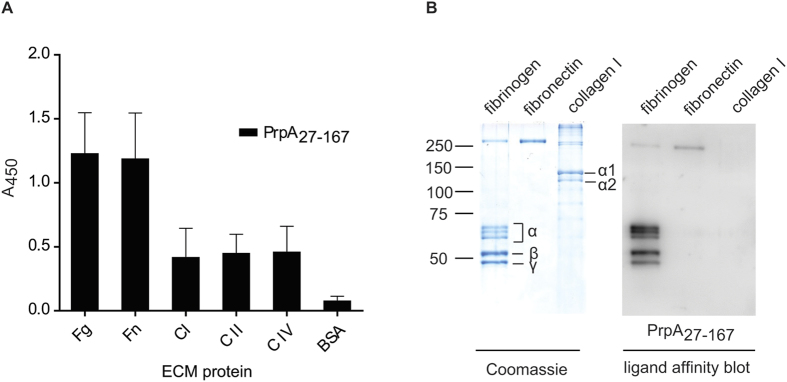
Binding of PrpA to ECMs proteins. Panel A shows the binding of PrpA_27-167_ to immobilized extracellular matrix (ECM) proteins fibrinogen (Fg), fibronectin (Fn) and collagen type I (C I), II (C II) and IV (C IV) as determined by ELISA. These data are averages of two independent experiments performed with two technical replicates each. Panel B shows ligand-affinity blots of the binding of PrpA_27-167_ to ECMs proteins transferred to nitrocellulose membranes. Binding of PrpA_27-167_ to ECMs proteins was detected using anti-PrpA IgG and HRP-goat anti-rabbit IgG. ECM proteins were also visualized after SDS – PAGE by staining with Coomassie. The different subunits of fibrinogen, collagen I and marker sizes (in kDa) are indicated.

In Gram-positive bacteria, surface-exposed proteins can mediate adherence to host tissues, often involving interactions with ECM components^40^,^41^,^42^. Therefore we studied whether PrpA could have a role in the adhesion to ECM proteins. Using ELISA, we found that PrpA_27-167_ binds to immobilized fibrinogen and fibronectin (Fig. 7A). Binding of PrpA_27-167_ to collagen types I, II and IV was also observed but at approximately three-fold lower levels compared to binding to fibrinogen and fibronectin. By ligand affinity blotting, we confirmed that PrpA_27-167_ binds to fibrinogen and fibronectin (Fig. 7B), but binding to collagen I could not be demonstrated (Fig. 7B) and identical, negative results were observed for collagen II and collagen IV (data not shown). Based on these data, we conclude that, among the tested ECM proteins, the N-terminal domain of PrpA only mediates binding to fibrinogen and fibronectin.

Binding of *E. faecium* E1162 and Δ*prpA* to fibrinogen and fibronectin was also assessed by ELISA. E1162 was able to bind both fibrinogen and fibronectin however no significant difference between wild-type and mutant was found (data not shown), which could be due to the functional redundancy of multiple fibrinogen binding proteins in *E. faecium*^43^.

### PrpA_27-167_ binds to human platelets

**Figure 8:**
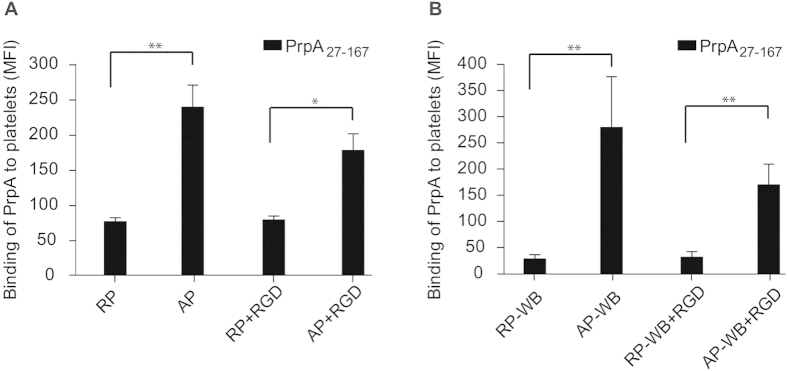
Binding of PrpA_27-167_ to platelets. Binding of the protein to platelets was measured by flow cytometry. PrpA_27-167_ was labeled with FITC. Binding of PrpA_27-167_ to resting (RP) and TRAP-activated washed platelets (AP) is shown in panel A and to resting (RP-WB) and TRAP-activated platelets (AP-WB) in whole blood in panel B. In panels A and B, D-arginyl-glycyl-L-aspartyl-L-tryptophane (RGD) was used to block the fibrinogen receptor (GPIIb-IIIa α_IIb_β_3_) in resting (RP + RGD) and activated (AP + RGD) washed platelets and in resting (RP-WB + RGD) and activated platelets (AP-WB + RGD) in whole blood. The mean fluorescence intensity (MFI) is shown. The figures represent data from three independent experiments and the error bars indicate the standard deviation. Data were analyzed by Student’s t-test and significant differences are indicated by one (*p* < 0.05) or two (*p* < 0.01) asterisks.

Apart from acting as extracellular matrix components, fibrinogen and fibronectin are also found in soluble forms in blood where they play an important role in the coagulation cascade^25^. Since the N-terminal domain of PrpA was found to mediate binding to fibrinogen, we examined the possibility whether PrpA_27-167_ could interact with platelets. The binding of FITC-labeled PrpA_27-167_ to resting platelets, and platelets that were activated using the thrombin receptor-activating peptide TRAP, was assayed by flow cytometry. These experiments were performed with washed platelets, thereby minimizing the amount of fibrinogen in the assay, and with resting and activated platelets in whole blood (RP-WP and AP-WB, respectively). Levels of surface-bound fibrinogen in each condition are shown in Fig. S3A for washed platelets and in Fig. S3B for platelets in whole blood. Activation of the washed platelets (Fig. S3C) and platelets in whole blood (Fig. S3D) was measured by the detection of the activation marker P-selectin on the surface of the platelets. PrpA_27-167_ was able to bind to RP (Fig. 8A) and to RP-WB (Fig. 8B), but activation of platelets considerably increased binding of PrpA_27-167_ to platelets, resulting in 3.0-fold higher binding to AP and 10.8-fold to AP-WB, compared to resting platelets in both conditions (Fig. 8A,B).

The increased binding of PrpA_27-167_ to activated platelets could be due to the increase in surface area of platelets upon activation^44^,^45^. Alternatively, binding of PrpA_27-167_ to activated platelets may be mediated by the release and subsequent binding of fibrinogen to the platelet surface during activation, which would reflect the ability of PrpA to bind this ECM protein. Blocking the fibrinogen receptor α(IIb)β3 on the platelet surface using D-arginyl-glycyl-L-aspartyl-L-tryptophane (RGD)^46^, did not affect binding of PrpA_27-167_ to RP or RP-WB. Blocking the fibrinogen receptor upon platelet activation (AP + RGD and AP-WB + RGD) decreased, but did not abolish, binding of PrpA_27-167_ to platelets (Fig. 8A,B). In AP + RGD, no fibrinogen was detected on the platelet surface (Fig. S3A), but binding of PrpA_27-167_ to the platelet surface was still observed. These observations suggest that both direct and fibrinogen-mediated interactions of PrpA_27-167_ contribute to binding of this protein to platelets.

The ability of *E. faecium* E1162 cells and the *prpA* deletion mutant to bind to platelets was also assayed. Both E1162 and the *prpA* deletion mutant were able to bind to platelets but no differences were observed (data not shown). This result probably reflects redundancy of multiple platelet-binding proteins that are important for the interaction of *E. faecium* with platelets, as previously described for *E. faecalis*^29^,^47^,^48^.

## Discussion

*E. faecium* is a commensal of the gastrointestinal tract but due to the accumulation of antibiotic resistance determinants and other adaptive elements, it has become an important nosocomial pathogen^3^,^5^,^39^,^49^. For opportunistic pathogens like *E. faecium*, which live in a wide variety of ecological niches ranging from plants and insects to the gastrointestinal tract of mammals, temperature is a particularly important environmental cue^1^,^30^,^50^. Under these different conditions, *E. faecium* needs to adjust its gene expression to survive in the environment and to be successful when interacting with the host^31^. Surface-exposed proteins in enterococci can facilitate bacterial interactions with host factors^9^, that might become exposed due to the use of indwelling medical devices in hospitalized patients. In the present study, we describe a thermo-regulated surface-exposed protein in *E. faecium*, termed PrpA, of which the N-terminal domain interacts with ECM components.

The presence of the C-terminal LPKSG motif in PrpA is predicted to allow anchoring of this protein to peptidoglycan. We hypothesized that in its peptidoglycan-anchored form, the proline-rich repeat region of PrpA may adopt a poly-proline helix-like conformation, similar to surface proteins with proline-rich repeat regions of streptococci^35^,^51^, thereby extending the functional N-terminal domain to the exterior milieu. For this reason, we focused on the function of the N-terminal domain of PrpA. However, the exact contribution of the C-terminal domain still needs to be investigated.

In accordance with the prediction that PrpA is a surface protein, we were able to detect surface-exposed PrpA on *E. faecium* E1162 cells, at higher levels when cultured at 37 °C than at 25 °C. *E. faecium* strains originating from healthy individuals that were previously assigned to clade B^39^, did not produce PrpA under the conditions tested. On the other hand, strains from clade A^39^, which covers strains of clinical and animal origins, can produce PrpA in a thermo-regulated fashion. Strains from *E. faecium* clade A are genetically distinct from the human commensal clade B strains and have been following distinct evolutionary trajectories^39^. In clade B strains, the role of PrpA remains to be determined and the expression of *prpA* may be regulated by environmental triggers other than temperature.

Using ELISA and ligand affinity blotting, we showed that the N-terminal domain of PrpA (PrpA_27-167_) binds to fibrinogen and fibronectin. We also observed that PrpA_27-167_ is able to interact with activated and resting platelets. Upon activation, fibrinogen bound to the platelet surface seems to facilitate the interaction of PrpA_27-167_ with the platelets, congruent with the ECM-binding characteristic of this protein. However, the binding of PrpA_27-167_ to activated washed platelets upon treatment with RGD, blocking the fibrinogen receptor, suggests that PrpA_27-167_ can also bind directly to platelets, in a fibrinogen-independent manner as has previously been described for other bacterial proteins^29^,^47^,^48^. The observation that deletion of *prpA* does not impair binding of *E. faecium* E1162 to fibrinogen, fibronectin and platelets, could reflect the functional redundancy of surface-exposed proteins of *E. faecium* in binding ECM components^15^,^27^,^28^.

Surface proteins of *E. faecium* are studied to understand the roles these proteins play in mediating interactions between the bacterial cell and its environment. PrpA is a surface protein that is characteristic for *E. faecium* and closely related enterococci, as it contains a unique N-terminal domain. In this study, we show that the N-terminal domain of PrpA interacts with fibrinogen, fibronectin and platelets. The thermo-regulated production of PrpA by clinical *E. faecium* isolation suggests that this protein may have a specific, but as yet undetermined, role in the colonization and infection of mammals.

## Methods

### Bacterial isolates, construction of a markerless *prpA* deletion mutant and *in trans* complementation

Information on the strains and plasmids that were used in this study is provided in Table S2. *E. faecium* was grown in Brain Heart Infusion (BHI; Oxoid, Basingstoke, United Kingdom) broth, unless otherwise noted. *Escherichia coli* was grown in Luria-Bertani Broth (LB; Oxoid). When needed, appropriate antibiotics were used at the following concentrations: gentamicin 300 μg ml^−1^ for *E. faecium* and 25 μg ml^−1^ for *E. coli*, spectinomycin 300 μg ml^−1^ for *E. faecium* and 100 μg ml^−1^ for *E. coli*, and erythromycin 25 μg ml^−1^ for *E. faecium* and 150 μg ml^−1^ for *E. coli*. All antibiotics were obtained from Sigma-Aldrich (Saint Louis, MO, USA).

To generate a markerless *prpA* deletion mutant in *E. faecium* E1162, we used a previously described technique^52^. In brief, the 5′ and 3′ flanking regions (approximately 500 bp each) of the *prpA* gene were PCR amplified with two sets of primers: Up-PrpA-F-XhoI and Up-PrpA-R- EcoRI at the 5′ end, and down-PrpA-F- EcoRI and down-PrpA-R-SmaI at the 3′ end of *prpA* (primer sequences are listed in Table S3). The two flanking regions were then fused together by PCR and cloned into pWS3^53^. A gentamicin-resistance cassette flanked by *lox66* and *lox71* sites was PCR amplified (oligonucleotides listed in Table S3) and cloned into the EcoRI site that was generated between the 5′ and 3′ flanking regions of *prpA* in the pWS3 construct as described previously^52^. Finally, the construct, named pMP1, was electrotransformed into *E. faecium* E1162 and a markerless deletion of *prpA* (termed Δ*prpA*) was generated as described before^52^.

For *in trans* complementation of Δ*prpA*, *prpA* with its native promoter was amplified by PCR using Accuprime High Fidelity Taq Polymerase (Life Technologies, Bleiswijk, The Netherlands) with primers Comp-prpA-F-BamHI and Comp-prpA-R-PstI, which introduced BamHI and PstI restriction sites. The resulting product was cloned into pMSP3535^54^. The construct was sequenced to confirm the absence of mutations in *prpA*. This plasmid was electrotransformed into Δ*prpA* as described previously^52^, generating the complemented strain Δ*prpA* + *prpA.* The expression of *prpA* in Δ*prpA* + *prpA* is under the control of its native promoter, as the inducible promoter on pMSP3535 is not activated by the addition of nisin in these experiments.

### Determination of growth curves

*E. faecium* E1162, its isogenic Δ*prpA* mutant, the *in trans* complemented Δ*prpA* + *prpA* strain and E1162 carrying the vector pMSP3535^54^, which was used for complementation, were grown overnight at 37 °C and 25 °C in BHI containing appropriate antibiotics. Cultures were diluted 1:50 in pre-warmed BHI at the appropriate temperature and the A_660_ was recorded for every 30 min until stationary phase was reached. Each experiment was performed in triplicate.

### Transcriptome analysis of *E. faecium* E1162

To determine thermo-regulated gene expression, RNA was isolated from *E. faecium* E1162 grown in BHI until the mid-exponential growth phase (A_660_ = 0.3) at 25 °C and 37 °C in a shaking (200 rpm) water bath. Further details of growth conditions, RNA isolation, cDNA synthesis and labeling, microarray hybridization and data analysis have been described previously^52^.

Microarray data have been deposited in ArrayExpress (http://www.ebi.ac.uk/arrayexpress) under accession number E-MEXP-3941.

### Quantitative real-time RT-PCR (qRT-PCR) analysis of *prpA* expression

Total RNA was isolated as described before and used to confirm the transcriptome analysis by qRT-PCR^55^. cDNA was synthesized using the Superscript III First-Strand Synthesis System (Life Technologies, Breda, The Netherlands) according to the manufacturer’s instructions. Using synthesized cDNAs, qRT-PCR was performed using the Maxima SYBR Green/ROX qPCR Master Mix (Thermo Scientific, Breda, The Netherlands) and a StepOnePlus instrument (Life Technologies). The expression of *tufA* was used as a housekeeping control. Ct values were calculated using the StepOne analysis software v2.2. REST 2009 V2.0.13 (Qiagen, Venlo, The Netherlands) was used to determine the transcript levels, relative to *tufA,* of the assayed genes and for statistical analysis. We also performed qPCRs on reaction mixtures that lacked reverse transcriptase. In these negative control samples Ct values were consistently higher (>34) than in the samples in which reverse transcriptase was added (Ct values ranging between 15 and 26), indicating that residual or contaminant DNA was present at minimal quantities and did not influence the determination of gene expression levels by qPCR. This experiment was performed with four biological replicates.

### Determination of the 5′ end of the *prpA* transcript

We used 5′RACE (Life Technologies) to map the 5′-end of the *prpA* mRNA, following the manufacturer’s instructions using two *prpA*-specific primers (GSP1 and GSP2) (Table S3). The amplified product was cloned using the CloneJET PCR cloning kit (Thermo Scientific) and sequenced by Sanger sequencing.

### Heterologous overexpression and purification of PrpA

A gene fragment of *prpA*, encoding the N-terminal domain of PrpA excluding the N-terminal signal sequence, was amplified with the primers PrpA-BamHI and PrpA_27-167_-R-NotI-STOP. The purified protein is referred to in this manuscript as PrpA_27-167._ The PCR products were digested with BamHI and NotI and then cloned into the similarly digested overexpression vector pEF110^56^, resulting in pMP2 which is an overexpression construct encoding the proteins with an N-terminal polyhistidine tag. The overexpression construct was transformed into *E. coli* BL21 (DE3) and further overexpression and purification of the recombinant proteins was performed as described previously^56^.

### Production of anti-PrpA polyclonal antibodies

Polyclonal antibodies against PrpA were raised by Eurogentec (Belgium) according to their rabbit immunization protocol. From the rabbit serum, IgG was purified using a 1-ml HiTrap Protein G HP column (GE Healthcare, Zeist, The Netherlands) and dialyzed overnight in phosphate buffered saline (PBS; 138 mM NaCl, 2.7 mM KCl, 140 mM Na_2_HPO_4_, 1.8 mM KH_2_PO_4_, adjusted to pH 7.4 with HCl).

### Quantification of surface-exposed PrpA by flow cytometry

Levels of surface-exposed PrpA were determined by flow cytometry, essentially as described previously^57^ with some minor modifications. In brief, *E. faecium* strains were grown overnight in BHI at 37˚C and 25˚C. The cells were then diluted (1:50) in pre-warmed media and cultured further at the appropriate temperature. Samples of the cultures were taken during different time points of the growth curve (A_660_ = 0.3, 0.5, 0.7, 1.0) and pelleted by centrifugation at 6,500 *g* for 1 min. PrpA was detected using 1:100 anti-PrpA rabbit IgG and 1:50 FITC-labeled goat anti rabbit IgG (Sigma-Aldrich). Flow cytometry was performed using the FACS Calibur system (BD Biosciences, Breda, The Netherlands). The geometric mean fluorescence was used as a measure for cell surface-exposed PrpA. This experiment was performed with three biologically independent replicates and statistical analysis of the data was performed using a two-tailed Student’s t-test.

### Sequence analysis of *prpA* in *E. faecium* strains

The *prpA* gene of different *E. faecium* strains was amplified by PCR and sequenced using the primers PrpA_Fw and PrpA*_*Rv (Table S3). Analysis of the sequences was performed using Serial Cloner version 2.6 and alignments were made using Clustal Omega (http://www.ebi.ac.uk/Tools/msa/clustalo/). The sequences have been deposited in Genbank under the following accession numbers: KP030673 (E980), KP030674 (E1007), KP030675 (E1071), KP030676 (E1574), KP030677 (E1575), KP030678 (E1578), KP030679 (E1636), KP030680 (E1644), KP030681 (E1731), KP030682 (E1972), KP030683 (E2560), KP030684 (E2620), KP030685 (E3548), KP030686 (E7345).

### Determination of PrpA localization by transmission electron microscopy (TEM)

The cellular localization of PrpA in *E. faecium* E1162 was determined by transmission EM with immunogold labeling as described previously^58^. Bacteria were grown to mid-exponential phase (A_660_ = 0.3) and PrpA was detected using purified anti-PrpA rabbit IgG labeled with 1:60 diluted Protein A-gold (10 nm). Microscopy was performed on a JEOL 1010 transmission electron microscope (JEOL Europe, Nieuw-Vennep, The Netherlands).

### ELISAs to determine binding of PrpA to ECM components

ELISAs to determine binding of PrpA_27-167_ to ECM components were performed as previously described^15^. After coating with ECM proteins, 50 μl of a 25 μg/ml solution in PBS of PrpA_27-167_ was used in the binding experiment. All ECMs proteins were obtained from Sigma-Aldrich. Binding of PrpA to ECM proteins was detected by anti-PrpA IgG (1:1000) and peroxidase-conjugated goat anti-rabbit IgG-horseradish peroxidase 1:10000 from Southern Biotech (Birmingham, AL; USA) and measuring absorbance at 450 nm.

### Ligand affinity blotting

Ligand affinity blotting was carried out as described by Hendrickx *et al.*^15^. 1 μg of ECM protein was loaded per well onto a 7.5% SDS gel. PVDF membranes (Merck Millipore) were incubated with purified PrpA_27-167_ at a concentration of 1 nM.

### FITC labeling of PrpA

FITC was dissolved at a concentration of 1 mg/ml in 1 M sodium carbonate buffer pH 9.6. A 2 mg/ml solution of PrpA_27-167_ was prepared in the same buffer and 500 μl of this solution was mixed with 55 μl of FITC solution. This mixture was incubated for 2  h at 4 °C in the dark. After labeling, the solution was desalted using Polyacrylamide Spin Desalting Columns (Thermo Scientific). FITC labeling of the protein was assessed by SDS-PAGE, running both labeled and unlabeled protein onto a 12% gel and imaging the gel under the GFP channel of an Image Quant LAS4000. FITC was obtained from Sigma-Aldrich.

### Binding of PrpA_27-167_ to human platelets

Whole blood was collected from three healthy volunteers using vacuum blood collection system tubes containing 3.2% sodium citrate. Platelets were isolated and washed as previously described^59^. The protocol for blood collection was approved by the Institutional Review Board of the University Medical Center Utrecht. Written informed consent was obtained from all donors in accordance with the declaration of Helsinki. For each donor, the mean platelet volume (MPV) was measured using the cell analyzer CELL-DYN 1800 (Abbott) in both whole blood and in washed platelets (WP) to exclude that activation of the platelets had occurred during isolation. Platelets were left at room temperature for at least 30 min to ensure a resting state before they were used in the experiments.

In a final volume of 200 μl, FITC-labeled PrpA_27-167_ (50 μg/ml) were mixed with 20 μl of either whole blood or washed platelets (adjusted to 200,000 platelets/μl). Whole blood and platelets were activated with 200 μM of the thrombin receptor-activating peptide SFLLRN (TRAP-6) from Bachem (Bubendorf, Switzerland). Platelet activation was monitored through binding of phycoerythrin-labelled mouse anti-human P-selectin antibodies (diluted 1:50; BD Biosciences) to the activation marker P-selectin on the platelet surface^60^. Binding of fibrinogen to platelets was detected using FITC labeled rabbit anti-human fibrinogen antibodies (Dako-Agilent Technologies, Heverlee, Belgium, diluted 1:50). The fibrinogen receptor (α_IIb_β_3_) was blocked using 100 μM of D-arginyl-glycyl-L-aspartyl-L-tryptophane (RGD)^46^, synthesized at the Department of Membrane Enzymology, Faculty of Chemistry, Utrecht University (Utrecht, The Netherlands). All samples were incubated for 30 min at room temperature and then fixed in a 0.2% paraformaldehyde, 0.9% NaCl solution. Flow cytometric detection of the binding of PrpA_27-167_ to platelets was performed on a FACSCanto II system (BD Biosciences). Statistical analysis of the data was performed using a two-tailed Student’s *t*-test.

## Additional Information

**How to cite this article**: Guzmán Prieto, A. M. *et al.* The N-terminal domain of the thermo-regulated surface protein PrpA of *Enterococcus faecium* binds to fibrinogen, fibronectin and platelets. *Sci. Rep.* **5**, 18255; doi: 10.1038/srep18255 (2015).

## Supplementary Material

Supplementary Information
