# Supplementary material for: The N-terminal domain of the thermo-regulated surface protein PrpA of Enterococcus faecium binds to fibrinogen, fibronectin and platelets
Source: Sci Rep. 2015 Dec 17;5:18255. doi: 10.1038/srep18255 (PMC4682149; doi:10.1038/srep18255)
Supplement: Supplementary Information [file srep18255-s1.pdf]

Supplementary materials

The N-terminal domain of the thermo-regulated surface protein PrpA of *Enterococcus faecium* binds to fibrinogen, fibronectin and platelets

Ana M. Guzmán Prieto[1], Rolf T. Urbanus[2], Xinglin Zhang[1], Damien Bierschenk[1], Arnold Koekman[2], Miranda van Luit-Asbroek[1], Janneke P. Ouwerkerk[1], Marieke Pape[1], Fernanda Paganelli[1], Dominique Wobser[3], Johannes Huebner[4], Antoni P.A. Hendrickx[1], Marc J.M. Bonten[1], Rob J.L. Willems[1], Willem van Schaik[1].

[1] Department of Medical Microbiology, [2] Department of Clinical Chemistry and Hematology, University Medical Center Utrecht, Utrecht, The Netherlands. [3] Department of Environmental Health Sciences, University Medical Center Freiburg, Freiburg, Germany. [4] Division of Pediatric Infectious Diseases, Hauner Children´s Hospital, Ludwig Maximilian University Munich, Munich, Germany.

Supplementary tables

Table S1: List of genes that exhibit higher expression during mid-exponential growth at 37°C than at 25°C as determined by transcriptome analysis.[a]

Locus tag	Annotation	Expression ratio 37°C / 25°C
EfmE1162_1194	hypothetical protein	4.5
EfmE1162_0376	surface protein, putative	4.4
EfmE1162_2382	hypothetical protein	3.2
EfmE1162_1492	tyrosine decarboxylase	3.2
EfmE1162_1491	antiporter	3.0
EfmE1162_1284	Fmu	2.9
EfmE1162_2341	conserved hypothetical protein	2.5
EfmE1162_1038	PspC domain family	2.4
EfmE1162_2340	ribosomal protein L32	2.4
EfmE1162_1737	conserved hypothetical protein	2.4
EfmE1162_2343	cobalamin synthesis protein/P47K family protein	2.3
EfmE1162_1490	Na+/H+ antiporter NhaC	2.3
EfmE1162_1702	conserved hypothetical protein	2.3
EfmE1162_0259	DltD protein	2.3
EfmE1162_0769	hypothetical protein	2.3
EfmE1162_2266	methionine gamma-lyase	2.2
EfmE1162_2342	ribosomal protein L33	2.2
EfmE1162_0678	hypothetical protein	2.2
EfmE1162_1816	branched-chain amino acid transport protein AzlD	2.2
EfmE1162_1815	AzlC family protein	2.2
EfmE1162_0261	protein DltB	2.1
EfmE1162_1037	conserved hypothetical protein	2.1
EfmE1162_1704	collagen adhesin	2.1
EfmE1162_0446	hypothetical protein	2.1
EfmE1162_2327	manganese transport system ATP-binding protein MntA	2.1
EfmE1162_0770	conserved hypothetical protein	2.1
EfmE1162_0644	shikimate kinase	2.1
EfmE1162_0260	D-alanyl carrier protein	2.1
EfmE1162_0263	putative D-Ala-teichoic acid biosynthesis protein	2.1
EfmE1162_0601	hydrolase	2.0
EfmE1162_2339	30S ribosomal protein S14 1	2.0
EfmE1162_2639	hypothetical protein	2.0
EfmE1162_1521	hypothetical protein	2.0
EfmE1162_0318	hypothetical protein	2.0

[a] The genes which exhibit a differential expression >2 between 37°C and 25°C are shown here.

Table S2: Strains and plasmids used in this study

Strain or plasmid	Relevant characteristic(s)	Source
E. faecium		
E1162	Clinical isolate (blood infection); Amp^r, Tet^r	33
Δ*prpA*	Markerless deletion mutant of *prpA* in E1162	This study
Δ*prpA*+*prpA*	*In trans* complementation strain of Δ*prpA*, Δ*prpA* harboring pMSP3535-*prpA*	This study
Aus0004	Clinical isolate (bloodstream infection)	61
E1644	Hospital outbreak, identical to EnGen0051	39
E2560	Clinical isolate (bloodstream infection), identical to strain EnGen0046	39
E1731	Clinical isolate (bloodstream infection), identical to strain EnGen0036	39
E1071	Hospital surveillance (feces)	33
E1636	Clinical isolate (bloodstrean infection)	33
E1574	Isolated from feces of a dog, identical to strain EnGen0020	39
E1575	Isolated from feces of a chicken, identical to strain EnGen0001	39
E1578	Isolated from feces of a mini pig, identical to strain EnGen0007	39
E2620	Clinical isolate (bloodstream infection), identical to strain EnGen0038	39
E980	Isolated from feces of a non-hospitalized individual	33
E1972	Clinical isolate (bloodstream infection), identical to strain EnGen0033	39
E1007	Isolate from feces of a non-hospitalized individual, identical to strain EnGen0015	39
E3548	Clinical isolate (bloodstream infection), identical to strain EnGen0047	39
E. coli		
BL21 (DE3)	Strain for recombinant protein expression	Invitrogen
Plasmids		
pEF110	Overexpression vector for *E. coli*, contains a sequence for an N-terminal histidine tag	Laboratory stock
pWS3	Gram-positive thermosensitive origin; Spc^r	53
pMP1	pWS3 derivative containing fused 5'and 3'flanking regions of *prpA*	This study
pMP2	pEF110 derivative carrying constructs for overexpression of $PrpA_{27\text{-}396}$ or $PrpA_{27\text{-}167}$	This study
pWS3-Cre	Derivative of pWS3 expressing *cre* recombinase in *E. faecium*	52
pMSP3535	pAMβ1 (from pIL252), ColE1 replicon, Erm^r, nisRK, PnisA	54
pMSP3535-*prpA*	Plasmid for the complementation of Δ*prpA*, pMSP3535 carrying *prpA*	This study

Amp: ampicillin; Tet: Tetracycline; Spc: Spectinomycin; Erm: Erythromycin; nisRK: nisin two-component system; PnisA: nisin inducible promoter

Table S3: Oligonucleotides used in this study

Primer	Sequence (5’ – 3’)
Up-PrpA-F-XhoI	CCGCTCGAGCGAATTTGTCTGACAGCTGAA
Up-PrpA-R-EcoRI	GATTTTGGTAGAATTCGATAAGCTTAAGGCTGCAAA
Down-PrpA-F-EcoRI	AGCTTATCGAATTCTACCAAAATCCGGAGAGTCACAA
Down-PrpA-R-SmaI	GTCGCCCGGGGGCGTTTGGGGCTTGATTGTCGG
Comp prpA F BamHI	ACGGGATCCCGATGTTCAGATAGGAAAAGAAAAA
Comp prpA R PstI	CAACTGCAGTTGGGGTCGTTTTCTGGATA
PrpA check dn	GAGGCATCCGAGATGTTGTT
PrpA check up	AAACCGTACAATGCTAAAAATGC
PrpA_Fw	AATCAGACAGTCCACACAGAG
PrpA_Rv	AATGATTCCGCTCCACAGTA
PrpA-BamHI	GTGGGGATCCGAGGAGATAGAAACAGCCAAATGG
$PrpA_{27-167}$-R-NotI-STOP	GGTCGCGGCCGCCTAAACAAGATCTCCTCCTGAGTC
pAT392_EcoRI_lox66_genta_F	GAGGGAATTCTACCGTTCGTATAGCATACATTATACGAAGTTATGATAAAC CCAGCGAACCATTTGAGG
pAT392_EcoRI_lox71_genta_R	CTCCGAATTCTACCGTTCGTATAATGTATGCTATACGAAGTTATTCAATCTT TATAAGTCCTTTTATAA
qPCR Fw E1162_1194	CGACCACTTTGAAAACCCAGA
qPCR Rv E1162_1194	TCTCTTTGAGTTTCTGATCCATTGC
qPCR Fw E1162_0376	AATCGACAAAGCGGGATCAA
qPCR Rv E1162_0376	CAGCGACCATCAAAGCAAAA
qPCR Fw E1162_2382	TTTCCCATGCTGATTCGACA
qPCR Rv E1162_2382	CTACGCCAGCAAGTGTGTGC
qPCR Fw E1162_1492	CCAATCGCTTTTTCCAGCAA
qPCR Rv E1162_1492	CTTTGGTATGCCCGCAACAT
GSP1	CGTCTGGATTTTCTGTAGGCGG
GSP2	GGTTCTTCAACAAGATCTCCTCC

Supplementary figures

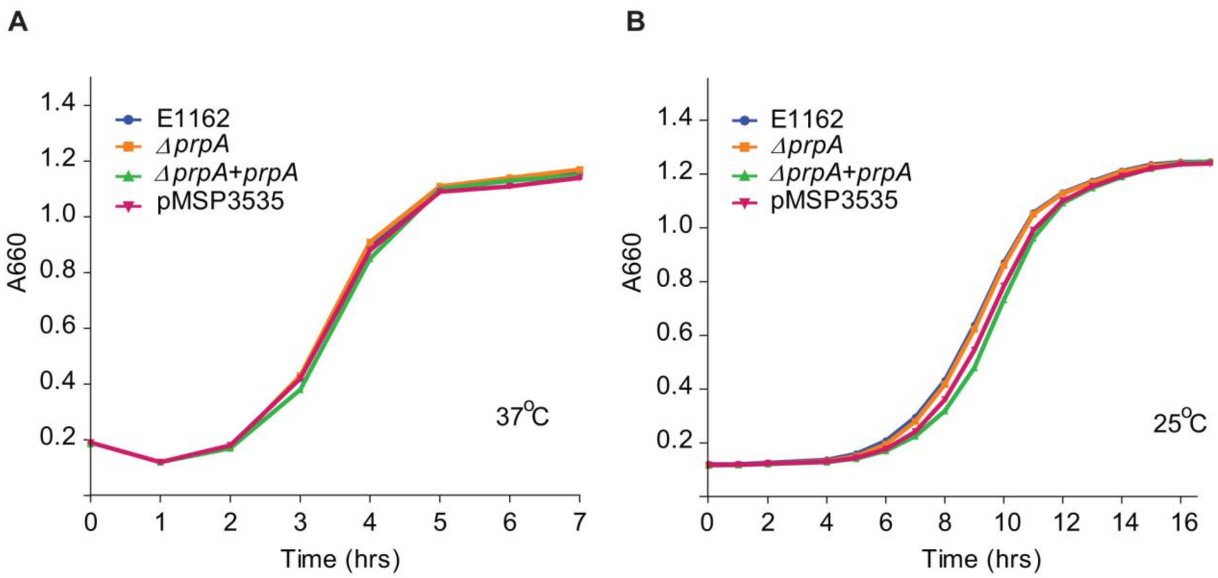

Fig. S1. Growth curves of *E. faecium* strains. Panel A and panel B show the growth curves for E1162, Δ*prpA*, Δ*prpA*+*prpA* and E1162 with the vector (pMSP3535) used for *in trans* complementation at 25°C and 37°C respectively. The growth curves represent the averages of three independent experiments.

```
           Aus0004   catgaaagaggctcctttttacttaaattgttaaaacttgaaaata
           E1162     catgaaagaggctcctttttacttaaattgttaaaacttgaaaata
Clade A1   E1644     catgaaagaggctcctttttacttaaattgttaaaacttgaaaata
           E2560     catgaaagaggctcctttttacttaaattgttaaaacttgaaaata
           E1731     catgaaagaggctcctttttacttaaattgttaaaacttgaaaata
           E1071     catgaaagaggctcctttttacttaaattgttaaaacttgaaaata
           E1636     catgaaagaggctcctttttacttaaattgttaaaacttgaaaata
Clade A2   E1574     catgaaagaggctcctttttacttaaattgttaaaacttgaaaata
           E1575     catgaaagaggctcctttttacttaaattgttaaaacttgaaaata
           E1578     catgaaagaggctcctttttacttaaattgttaaaacttgaaaata
           E1007     catgaagggcgctcctttttacttaaattgttaaaactagaaaata
           E3548     catgaagggcgctcctttttacttaaattgttaaaactagaaaata
Clade B    E2620     catgaagggcgctcctttttacttaaattgttaaaacttgaaaata
           E980      catgaagggcgctcctttttagttaaattgttaaaacttgaaaata
           E1972     catgaagggcgctcctttttagttaaattgttaaaacttgaaaata
                     ****** *  *********** ***************** *******

           Aus0004   ttactgtttttttcataaatttagacaaaaaaacagtaaacatata
           E1162     ttactgtttttttcataaatttagacaaaaaaacagtaaacatata
Clade A1   E1644     ttactgtttttttcataaatttagacaaaaaaacagtaaacatata
           E2560     ttactgtttttttcataaatttagacaaaaaaacagtaaacatata
           E1731     ttactgtttttttcataaatttagacaaaaaaacagtaaacatata
           E1071     ttactgtttttttcataaatttagacaaaaaaacagtaaacatata
           E1636     ttactgtttttttcataaatttagacaaaaaaacagtaaacatata
Clade A2   E1574     ttactgtttttttcataaatttagacaaaaaaacagtaaacatata
           E1575     ttactgtttttttcataaatttagacaaaaaaacagtaaacatata
           E1578     ttactgtttttttcataaatttagacaaaaaaacagtaaacatata
           E1007     ttactgtttttttcatgaatctagacaaaaagacagtaaacatata
           E3548     ttactgtttttttcatgaatctagacaaaaagacagtaaacatata
Clade B    E2620     ttactgtttttttcatgaatctagacaaaaagacagtaaacatata
           E980      ttactgtttttttcatgaaactagacaaaaagacagtaaacatata
           E1972     ttactgtttttttcatgaaactagacaaaaagacagtaaacatata
                     **************** **  ********** **************

           Aus0004   ggtagaatctctactatcttatttcttgcatacaaaaaaatcaaggagtgagagagttatg
           E1162     ggtagaatctctactatcttatttcttgcatacaaaaaaatcaaggagtgagagagttatg
Clade A1   E1644     ggtagaatctctactatcttatttcttgcatacaaaaaaatcaaggagtgagagagttatg
           E2560     ggtagaatctctactatcttatttcttgcatacaaaaaaatcaaggagtgagagagttatg
           E1731     ggtagaatctctactatcttatttcttgcatacaaaaaaatcaaggagtgagagagttatg
           E1071     ggtagaatctctactatcttatttcttgcatacaaaaaaatcaaggagtgagagagttatg
           E1636     ggtagaatctctactatcttatttcttgcatacaaaaaaatcaaggagtgagagagttatg
Clade A2   E1574     ggtagaatctctactatcttatttcttgcatacaaaaaaatcaaggagtgagagagttatg
           E1575     ggtagaatctctactatcttatttcttgcatacaaaaaaatcaaggagtgagagagttatg
           E1578     ggtagaatctctactatcttatttcttgcatacaaaaaaatcaaggagtgagagagttatg
           E1007     ggtacaatctctactatcttatttcttgcatacaaaaaaatcaaggagtgagagagttatg
           E3548     ggtacaatctctactatcttatttcttgcatacaaaaaaatcaaggagtgagagagttatg
Clade B    E2620     ggcacaatctctactatcttatttcttgcatacaaaaaaatcaaggagtgagagagttatg
           E980      ggcacaatctctactatcttatttcttgcatacaaaaaaatcaaggagtgagagagttatg
           E1972     ggcacaatctctactatcttatttcttgcatacaaaaaaatcaaggagtgagagagttatg
                     ** * ********************************************************
```

Fig. S2. Alignment of the promoter region of *prpA* in different *E. faecium* strains. Fifteen *E. faecium* strains from different environments, clinical isolates (clade A1), animal strains (clade A2) and human commensals (clade B) were aligned using Clustal Omega (http://www.ebi.ac.uk/Tools/msa/clustalo/). The blue boxes indicated the inverted repeats. The red box depicts the transcriptional start site of *prpA*. Putative -35 and -10 promoter regions are shown in bold italics and the start of the *prpA* gene is underlined.

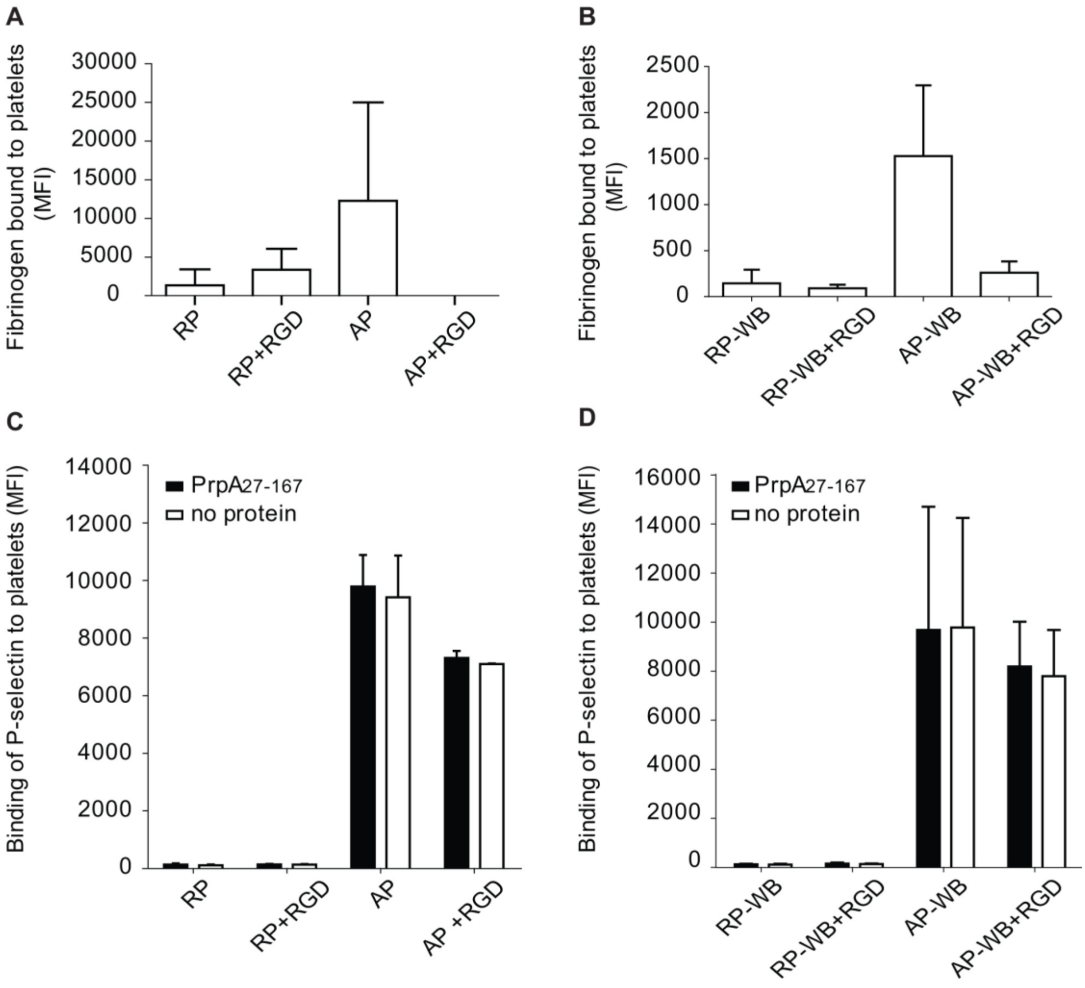

Fig. S3. Fibrinogen levels and platelet activation in washed platelets and in whole blood. Panel A shows the levels of fibrinogen bound to resting (RP) and TRAP-activated (AP) washed platelets and to resting (RP-WB) and TRAP activated platelets (AP-WB) in whole blood in panel B. Fibrinogen bound to platelets was measured by flow cytometry in the absence of PrpA using an α-fibrinogen antibody labeled with FITC. D-arginyl-glycyl-L-aspartyl-L-tryptophane (RGD) was used to block the fibrinogen receptor (GPIIb-IIIa) in resting (RP+RGD) and activated (AP+RGD) washed platelets and in resting (RP-WB+RGD) and TRAP activated platelets (AP-WB+RGD) in whole blood. Activation of platelets is demonstrated by the binding of P-selectin to the surface of washed platelets (panel C) and to platelets in whole blood (panel D). P-selectin was detected using phycoerythrin-labelled mouse anti-human P-selectin antibodies. For all panels, the mean fluorescence intensity (MFI) is shown. The results are data of three biological replicates.
